# Sensing Performance of Thermal Electronic Noses: A Comparison between ZnO and SnO_2_ Nanowires

**DOI:** 10.3390/nano11112773

**Published:** 2021-10-20

**Authors:** Matteo Tonezzer, Cristina Armellini, Laura Toniutti

**Affiliations:** 1 IMEM-CNR, Sede di Trento-FBK, Via alla Cascata 56/C, 38123 Trento, Italy; 2 Center Agriculture Food Environment, University of Trento/Fondazione Edmund Mach, Via E. Mach 1, 38010 San Michele all’Adige, Italy; 3 Department of Food Quality and Nutrition, Research and Innovation Centre, Fondazione Edmund Mach, Via E. Mach 1, 38098 San Michele all’Adige, Italy; 4 Institute for Photonics and Nanotechnologies (IFN)-National Research Council (CNR) CSMFO Lab, Via alla Cascata 56/C, 38123 Trento, Italy; cristina.armellini@unitn.it; 5 Fondazione Bruno Kessler (FBK)-Centro Materiali e Microsistemi (CMM), Via alla Cascata 56/C, 38123 Trento, Italy; 6 Agenzia Provinciale Protezione Ambiente, Settore Qualità Ambientale, U.O. Tutela dell’Aria e Agenti Fisici, Via Lidorno 1, 38123 Trento, Italy; laura.toniutti@provincia.tn.it

**Keywords:** metal oxide, gas sensor, resistive sensor, chemiresistor, electronic nose

## Abstract

In recent times, an increasing number of applications in different fields need gas sensors that are miniaturized but also capable of distinguishing different gases and volatiles. Thermal electronic noses are new devices that meet this need, but their performance is still under study. In this work, we compare the performance of two thermal electronic noses based on SnO_2_ and ZnO nanowires. Using five different target gases (acetone, ammonia, ethanol, hydrogen and nitrogen dioxide), we investigated the ability of the systems to distinguish individual gases and estimate their concentration. SnO_2_ nanowires proved to be more suitable for this purpose with a detection limit of 32 parts per billion, an always correct classification (100%) and a mean absolute error of 7 parts per million.

## 1. Introduction

Nowadays, the need for gas sensors is increasingly clear and important in many fields. Progress brings benefits but also hidden dangers, such as the effect of day-to-day breathing of harmful gases that ruin health. Air pollution in urban areas is only the most obvious of these dangers to human health [1,2,3]. Numerous industrial and artisanal processes emit gases with negative long-term effects [4,5]. Many materials used for objects that share the spaces inhabited by humans release volatile substances, the dangers of which are not yet known [6]. For these reasons, it is important to be able to monitor the presence of gas in environments and activities related to human life. The availability of tiny sensors that can be integrated into buildings, cars and portable devices, such as cell phones or even wearables, would allow for the creation of wide and capillary networks capable of monitoring the situation at a high level. Sensors of this type would also be important in many other fields, such as food and beverage quality [7,8], agriculture [9], security against terrorism [10] and early medical diagnosis [11]. Metal oxides (MOs) are the ideal candidate for small and cheap devices as they work very well as chemoresistors, i.e., electrical resistors, the value of which changes according to the surrounding atmosphere (the sensor only needs two electrodes, with very simple signal reading) [12,13,14].

Furthermore, the latest generation of chemoresistors use nanostructured materials, which have a very large surface that greatly increases the response intensity. The shape [15] and, more importantly, the dimensions [16] of the nanostructures influence the sensing performance of the sensors; therefore, one of the most used materials are nanowires (NWs). N-type semiconductors have been studied for longer and more thoroughly as they perform better as gas detectors [17,18]. The two materials most studied previously are SnO_2_ and ZnO, which are still the most used owing to their superior performance. Unfortunately, the fact that MOs are sensitive to a lot of gases is also a flaw as it is not possible to recognize what the sensor is detecting. In the same way, the simplicity of resistive sensors is also a defect, since the output signal they give is one dimensional and therefore intrinsically non-selective. The traditional solution to the lack of selectivity is to combine sensors based on different materials in an array, called an electronic nose [19]. This type of device has attracted a lot of interest as it shows a good balance between performance on the one hand and size and cost on the other [20,21]. Despite this, the cost and dimensions of a traditional electronic nose do not yet to allow for its widespread diffusion and integration into everyday devices. To this end, taking up Sysoev’s pioneering works [22,23], we have recently developed a new approach that uses the same nanostructured material working at different temperatures instead of different materials, as if it were an electronic nose [24,25,26]. The lower expected selectivity is balanced by the size (it can be a few square millimeters), which allows it to be easily integrated into a smartphone or smartwatch. Despite the great potential of this type of thermal electronic nose, its recent development means that its performance has not yet been studied in detail. In this work, we tested two different thermal electronic noses based on SnO_2_ and ZnO nanowires working at five different temperatures. The performance of the two systems was tested against five gases: acetone, ammonia, ethanol, hydrogen and NO_2_. The performance was evaluated in two steps: how well the electronic nose could distinguish the different gases and the error in the estimation of the gas concentration. The sensor based on ZnO nanowires classified 95% of the samples correctly, while the sensor based on SnO_2_ nanowires classified all gases perfectly (100%). The mean absolute error on the concentration estimate is low in both cases (7 parts per million for the SnO_2_ nanowires and 11 for the ZnO nanowires), but rises to 30 parts per million (ppm) in the case of the misclassified sample. Hence, SnO_2_ nanowires work best as active materials inside a thermal gradient based electronic nose. We emphasize that this preliminary work must be reconsidered according to the boundary conditions of the application for which the electronic nose is to be used.

## 2. Materials and Methods

### 2.1. Synthesis of SnO_2_ and ZnO Nanowires

Both tin oxide (SnO_2_) and zinc oxide (ZnO) nanowires were grown by means of chemical vapor deposition with modifications to the recipes in order to obtain nanowires of similar size. In both growth processes, an alumina vessel containing the source powder was placed inside a quartz tube in the center of a single-zone furnace (Lingdberg Blue M, Thermo Fisher Scientific, Waltham, MA, USA) at the highest temperature point. The substrate (a 1 × 3 cm^2^ rectangle of silicon wafer with 300 nm of thermally grown oxide and 5–7 nm of gold catalyst) was positioned downstream of the source at the optimum growth distance.

In the case of tin oxide nanowires, the source powder was 99.99% pure tin monoxide (Sigma-Aldrich, St. Louis, MO, USA), and the substrate was placed approximately 3 cm away from it. The quartz tube was pumped at 5 × 10^−3^ mbar and purged with high-purity argon (99.999%), and this process was repeated three times to clean the system. Next, the oven was heated up to 850 °C at a rate of 50 °C per minute and held at this temperature for five minutes. Finally, 0.35 standard cubic centimeters (sccm) of oxygen was flowed through the quartz tube, starting the 30 min growth process, after which the system was shut down and cooled naturally.

In the case of zinc oxide nanowires, the source powder was 99.995% pure zinc (Sigma-Aldrich, St. Louis, MO, USA), and the substrate was placed at about 7 cm from it. The quartz tube was cleaned as explained previously and then heated at a rate of 50 °C per minute to a temperature of 730 °C. After five minutes, a mixture of 50 sccm of argon and 2 sccm of oxygen was flowed for 40 min, then the process was finished and the system cooled naturally.

### 2.2. Material Characterization

The morphology of the tin oxide and zinc oxide nanowires was characterized with secondary electron microscopy (SEM) using a Hitachi S-4800 (Tokyo, Japan). The structure was investigated with X-ray diffraction (XRD) using a Philips Xpert Pro (Malvern Panalytical, Malvern, UK) operating at 40 kV with CuKα radiation.

### 2.3. Fabrication of the Sensor

To fabricate the sensor, the nanowires were transferred to another substrate, a 1 × 2 cm^2^ piece of silicon wafer with a 300 nm layer of thermally grown oxide. To perform this, each sample with the nanowires was sonicated in dimethylformamide for two seconds to obtain a dispersion, which was then deposited on the new substrate by spinning a few drops at 6000 rpm. On top of the substrate with the dispersed nanowires, a pair of interdigitated Ti/Pt electrodes with a thickness of 10/250 nm was then deposited by means of sputtering and UV lithography. The semiconductor nanowires acting as a bridge between the metal electrodes thus served as a chemoresistor.

### 2.4. Gas Sensor Measurements

Each interdigitated chemoresistive sensor was placed in the measurement chamber on top of a heatable sample holder. The measurement chamber was connected to gas cylinders through mass flow controllers in order to vary the type and concentration of gas to be tested. A pair of micromanipulators was used to contact the sensor electrodes and bring the signal to a multimeter (Keithely 2410, Cleveland, OH, USA) controlled with a homemade data acquisition program (LabView, National Instruments, Austin, TX, USA). The sensors were held at 500 °C for 6 h and powered with 1 V in order to stabilize the nanowires and their base resistance [27]. Both sensors showed a linear trend of current versus voltage, demonstrating good ohmic contact. Both sensors with SnO_2_ and ZnO nanowires were tested under the exact same conditions: at five different temperatures (200, 250, 300, 350 and 400 °C) towards the same five gases (acetone, ammonia, ethanol, hydrogen and nitrogen dioxide). Each gas was measured at nine different concentrations, ranging from 5 to 250 parts per million (ppm), in order to cover the concentrations indicated as dangerous by the institutions [28,29,30,31,32].

The definition S = R_AIR_/R_GAS_ was used to calculate the response intensity, where R_GAS_ and R_AIR_ are the resistance of the sensor in the presence of the target gas and in air, respectively. The standard definition was used to calculate the limit of detection (LoD): the intercept between the slope of the response as a function of concentration and three times the standard deviation of the signal.

### 2.5. Machine Learning

For each sensor, the response at the five temperatures, calculated as explained in the previous section, was combined into a five-dimensional point. Principal component analysis (PCA) [33] was applied to the five-dimensional points in order to reduce their dimensionality and visualize the relationships between the points relating to the various gases. The distinction of the different gases was instead carried out by a support vector machine [34] with a linear kernel used as a classifier, which worked on all five dimensions.

The points were then passed to five other support vector machines (SVM), one for each gas, depending on how they were classified in the previous step. These SVMs were used as five-dimensional regressors in order to estimate the gas concentration. It should be emphasized that for each point, the regressor relative to the gas that the system has previously classified was used, not the real one, as this can strongly increase the error in the concentration estimate.

## 3. Results and Discussion

### 3.1. Nanowire Characterization

**Figure 1:**
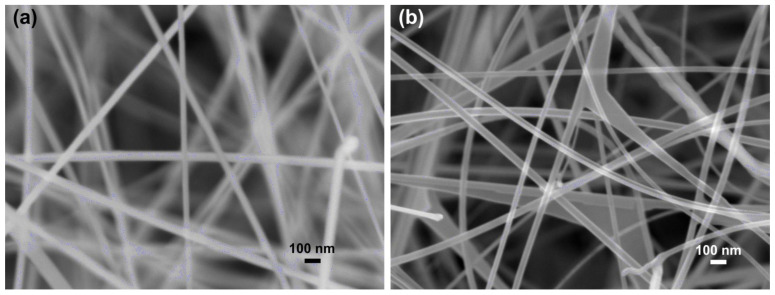
SEM images of the nanowires grown by CVD and used as gas sensors: (**a**) ZnO nanowires; (**b**) SnO_2_ nanowires.

The morphology of the zinc oxide and tin oxide nanowires obtained by chemical vapor deposition was studied with scanning electron microscopy; the SEM images are shown in Figure 1.

Figure 1a shows the ZnO nanowires which have an average diameter of about 50–60 nm and are very constant and homogeneous. Figure 1b instead shows the SnO_2_ nanowires, which have an average diameter of around 50–65 nm. In this case, the nanowires tend to create “sails” in some places, even if it is not a very frequent effect. The shape and size of the nanowires are quite similar, which allows for a better comparison of their performance as gas sensors.

**Figure 2:**
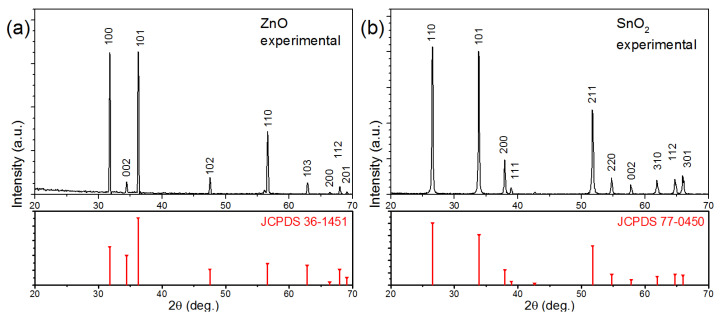
XRD patterns of the nanowires grown by CVD: (**a**) ZnO nanowires and (**b**) SnO_2_ nanowires. The top images show the experimental patterns (in black), while the bottom images show the reference pattern from ICDD (in red).

The structure of the nanowires was investigated with X-ray diffraction, shown in Figure 2. The experimental patterns obtained from the samples grown by CVD (before being transferred to fabricate the sensor) are shown in the upper row (in black), while in the bottom row (in red) the reference patterns from the International Center for Diffraction Data are shown.

It is evident that the experimental pattern in Figure 2a agrees with the underlying reference pattern, and each peak can be easily indexed to a hexagonal wurtzite with lattice parameters of a = b = 3.249 Å and c = 5.206 Å. Similarly, all the diffraction peaks present in the experimental pattern in Figure 2b can be easily indexed to the tetragonal phase of SnO_2_ with lattice parameters of a = b = 4.742 and c = 3.186 Å and therefore agree well with the standard values in the pattern below. In both experimental patterns, amorphous contributions and peaks from impurities or other phases are absent, confirming the high purity of the crystalline nanowires.

### 3.2. Gas Sensing Measurements

The sensor response was measured for each gas at the five temperatures (200–400 °C) in the traditional way, and the limit was calculated at each temperature. Since the sensor signal is louder at low temperatures, the LoD at 200 °C is the largest for each gas. For this reason, we considered this value as the limit of detection of the thermal electronic nose. The detection limits of the electronic nose based on ZnO nanowires were found to be 0.7, 2.1, 1.8, 1.5 and 0.9 parts per million respectively for acetone, ammonia, ethanol, hydrogen and nitrogen dioxide. The corresponding detection limits for the SnO_2_ nanowire-based electronic nose were 0.9, 1.2, 0.8, 0.2 and 0.4 parts per million. The average LoD is therefore 0.7 ppm for the ZnO nanowires and 1.4 ppm for the SnO_2_ ones. This also partly stems from the higher resistivity of the ZnO nanowires, which makes the signal noisier.

**Figure 3:**
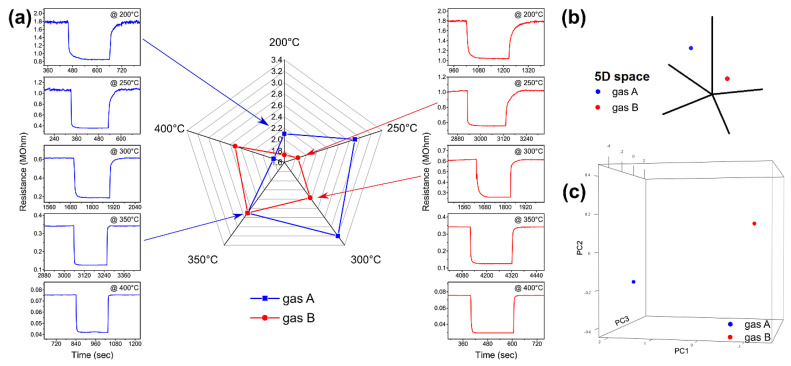
Procedure for combining the five sensor responses at different temperatures: (**a**) the responses calculated in the five graphs in each column (blue and red) are combined into a radar plot (blue and red, respectively); (**b**) the five response values become a five-dimensional point; (**c**) which, in our case, is reduced to three dimensional by PCA.

The five response values, calculated at the five temperatures according to the definition given in Section 2.4, were then combined together in five-dimensional points of this type: P_A_ = (R_A_^200°C^, R_A_^250°C^, R_A_^300°C^, R_A_^350°C^, R_A_^400°C^) and P_A_ = (R_B_^200°C^, R_B_^250°C^, R_B_^300°C^, R_B_^350°C^, R_B_^400°C^). A single response as a one-dimensional signal is inherently non-selective. Instead, the five responses combined contain a lot of information, not only the five values but also all the correlations between them. This can be seen in Figure 3a: if we compare the response of a sensor at a single temperature for gas A and for gas B (one of the plots in blue on the left and the corresponding in red in the right column), it is not possible to recognize the gas in question. If, on the other hand, we combine the five responses of the left column into the radar plot in blue in the center of Figure 3a and those of the right column into the radar plot in red, we can clearly see the difference, since the two forms relating to the two gases are clearly different.

The radar plot relative to a gas increases in amplitude as the concentration of the gas increases, but retains the same shape. It should be emphasized that radar plots are used only to be able to easily compare the responses of the thermal electronic nose to different gases: the machine learning algorithms work in 5D (using the points P_A_ and P_B_, like in Figure 3b). Unfortunately, it is impossible to compare too many overlapping shapes in a radar plot. Since 5D space is impossible to visualize, the best way to see relationships between responses related to different gases is to perform a principal component analysis, which reduces dimensionality (in our case from 5D to 3D or 2D) while keeping as much information as possible. Figure 3c shows how the two radar plots (blue and red) in Figure 3a appear in a PCA graph after reducing from five to three dimensions: each radar graph becomes a single point, which contains almost all the information of the five responses at different temperatures. In this way, it is possible to plot many points relative to different gases and clearly perceive the relationships between points of different gases or of the same gas at different concentrations. The greater the distance between the two points in the graph, the greater the measures from which they derive are different from each other.

### 3.3. Qualitative Dinstinction

**Figure 4:**
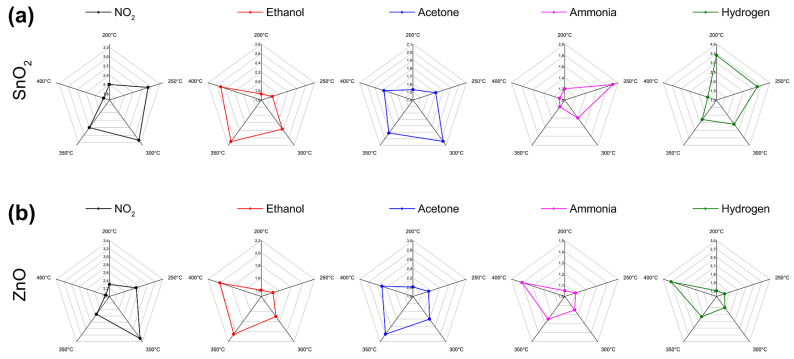
Radar plots obtained by measuring 100 ppm of each gas at five temperatures: (**a**) with the sensor based on SnO_2_ nanowires; (**b**) with the sensor based on ZnO nanowires.

As the first step, we plotted the responses of the sensors as radar plots to qualitatively evaluate how selective the two sensors are. The graphs obtained with the sensor based on SnO_2_ nanowires are shown in the upper row of Figure 4, while those obtained with the sensor based on ZnO nanowires are shown in the lower row.

As can be seen, the radar plots shown in the upper row of Figure 4 are quite different from each other and therefore seem to indicate a good selectivity of the sensor. On the bottom row, however, the last two radar plots look quite similar. In reality, the responses to hydrogen are much greater, as can be seen from the scale of the radar plot, but the selectivity (classification of the different gases) is based only on the shape and not on the size of the plot.

**Figure 5:**
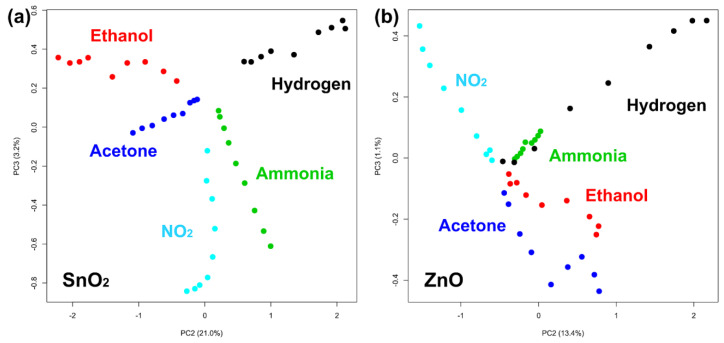
PCA plots showing measurements of the five gases obtained with (**a**) the sensor based on SnO_2_ nanowires and (**b**) the sensor based on ZnO nanowires.

Unfortunately, this method does not work well if one wants to compare different points of each gas because the graph becomes confusing with more than 3–4 plots. For this reason, we used the principal component analysis on the five-dimensional points obtained from the sensor responses, and the results are shown in Figure 5.

Although both methods (radar plots and PCA plots) are just approximations of the 5D situation, the PCA plots shown in Figure 5 can show many points more clearly. In Figure 5, the components PC2 and PC3 are shown since the component PC1 is mainly due to the variation in gas concentration; therefore, the figure shows the best point of view to appreciate the separation between the different target gases. The difference between the graph obtained with the SnO_2_ nanowires (left) and the one obtained with the ZnO nanowires (right) is remarkable since in the first graph the points relating to each gas seem clearly more separated.

In order to try to improve the distinction between gases, we attempted to normalize each group of five responses (each radar plot, or each point in the PCA) to its highest value. In this way, we expected the specificity of each gas (the shape of its radar plot) to be more important than the concentration in the comparison for the classification of the gas.

**Figure 6:**
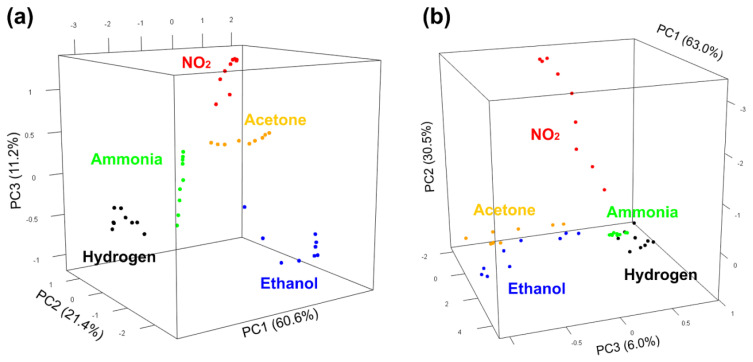
PCA plots showing the measurements after normalization obtained with (**a**) the sensor based on SnO_2_ nanowires and (**b**) the sensor based on ZnO nanowires.

In each image, the view that best separates the points of the different gases was chosen. As can be seen, the points in Figure 6a separate even better than in Figure 5a, demonstrating that normalization helps classification by reducing the contribution due to gas concentration. Unfortunately, the effect is minimal in Figure 6b, in which it seems that some gases are still not very separated.

However, it should be emphasized that these are only dimensional reductions of the true five-dimensional space, which is also limited by our visual perception. Hence, to have an objective vision that is not approximate and not distorted by human perception, we used machine learning algorithms that work directly in five dimensions.

### 3.4. Classification

A support vector machine, used as a classifier, was used as the “brain” for the distinction between gases. This machine learning algorithm is supervised, meaning it needs data to “learn” how to classify. For this reason, five different concentrations for each gas (5, 20, 50, 150 and 250 ppm) were used as the training dataset. Four other concentrations (10, 30, 100 and 200 ppm) were then used to test the performance of the sensing system.

The 5D points of the training set are used by the algorithm to identify hyperplanes (4D spaces) that divide the 5D space into zones related to each gas. Once this map has been created (as a sort of calibration), the next points are automatically compared with the map and classified according to the zone of 5D space they fall into.

The results of the classification obtained with the sensor based on SnO_2_ nanowires are shown in Table 1.

As can be seen from the confusion matrix in Table 1, all measured points were correctly classified; therefore, the sensor based on SnO_2_ nanowires perfectly distinguished the tested gases, albeit at different concentrations.

The same procedure was carried out with the five-dimensional points obtained from the responses of the sensor based on ZnO nanowires, and the results are reported in Table 2.

In this case, 19 out of 20 points are correctly classified, but one is misclassified. To be exact, the system mistakes the lowest concentration point of hydrogen for ammonia. This is in accordance with Figure 5b and Figure 6b, where the ammonia points are very close to the low concentration points relating to hydrogen.

It should be emphasized that this result is a much more advanced step than those in Section 3.2, since the thermal electronic nose in this case is able to distinguish gases autonomously, without a human having to observe any image and deduce anything. In fact, while the colors in Figure 5 and Figure 6 were given knowing a priori which gas it was, in this case, once trained, the electronic nose understands by itself which gas is being measured. The electronic nose based on ZnO nanowires correctly recognized the gas only in 95% of cases, while the one based on SnO_2_ nanowires recognized them all perfectly (100%).

### 3.5. Quantification

**Figure 7:**
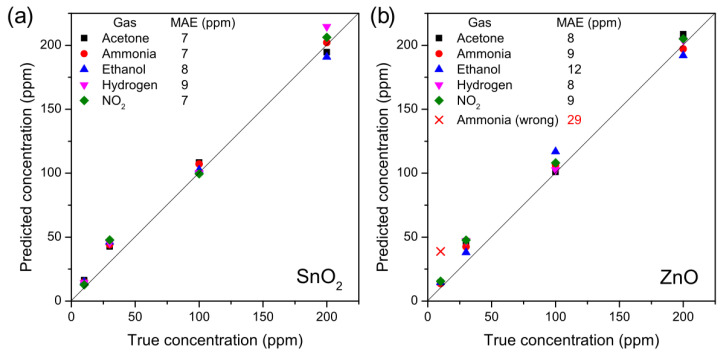
Predicted gas concentration as a function of the true concentration by the thermal electronic nose based on (**a**) SnO_2_ nanowires and (**b**) ZnO nanowires.

Once the gas was recognized with the classifier, the data were split based on the gas that the system recognized (right or wrong) and passed to another support vector machine. The five support vector machines, each related to a gas, worked as regressors in the five-dimensional space to estimate the gas concentration. The regression results are shown in Figure 7.

Figure 7a shows the estimated concentration from the thermal electronic nose based on SnO_2_ nanowires. The true concentration is on the abscissa, while on the ordinate there is the estimate of the electronic nose; therefore, the diagonal represents the perfect result. The estimates are all very close to the diagonal, with a very low error. The mean absolute error (MAE) is in fact less than 10 parts per million for all gases. Furthermore, at low concentrations, the estimates are always higher than the true concentration, and this is positive for the sensor: a possible threshold alarm would trigger with some false positives, but it would not risk tripping in the presence of gas.

The situation is worse in Figure 7b, which was obtained with the responses of the electronic nose based on the ZnO nanowires. In this case, the error on the estimate is generally greater, even if it remains good. The error increases significantly if we consider the wrongly classified measurement, since in this case 10 ppm of hydrogen was confused as 40 ppm of ammonia. It is therefore clear that classification is the most important step as it strongly influences the performance of gas concentration quantification.

## 4. Conclusions

The performance of two thermal electronic noses, identical in every respect except for the active material used as a sensor (ZnO and SnO_2_ nanowires, respectively), were studied regarding five gases (acetone, ammonia, ethanol, hydrogen and nitrogen dioxide). Nine different concentrations were measured for each gas from 5 to 250 ppm in order to test the performance along the entire concentration range around the hazard threshold. The electronic nose based on ZnO nanowires showed a correct classification in 95% of cases and an error that was influenced by the misclassifications. The detection system based on SnO_2_ nanowires, on the other hand, classified perfectly (100%) and estimated the concentration of all five gases with a mean absolute error of less than 10 ppm. The electronic nose based on SnO_2_ nanowires at different temperatures is therefore a good candidate for a gas detection system that is selective and small enough to be integrated into smartphones and other devices.

## Figures and Tables

**Table 1:** Confusion matrix obtained with the sensor based on SnO_2_ nanowires.

				Estimated		
		Acetone	Ammonia	Ethanol	Hydrogen	NO_2_
	Acetone	4				
	Ammonia		4			
**True**	Ethanol			4		
	Hydrogen				4	
	NO_2_					4

**Table 2:** Confusion matrix obtained with the sensor based on ZnO nanowires.

				Estimated		
		Acetone	Ammonia	Ethanol	Hydrogen	NO_2_
	Acetone	4				
	Ammonia		4		1	
**True**	Ethanol			4		
	Hydrogen				3	
	NO_2_					4

## Data Availability

The data presented in this study are openly available in Open Science Framework at doi:10.17605/OSF.IO/6D9MA.
